# Mutation of c.244G>T in *NR5A1* gene causing 46, XY DSD by affecting RNA splicing

**DOI:** 10.1186/s13023-021-02002-0

**Published:** 2021-08-30

**Authors:** Bingqing Yu, Yinjie Gao, Jiangfeng Mao, Xi Wang, Min Nie, Xueyan Wu

**Affiliations:** Department of Endocrinology, NHC Key Laboratory of Endocrinology (Peking Union Medical College Hospital), State Key Laboratory of Complex Severe and Rare Diseases, Peking Union Medical College Hospital, Peking Union Medical College, Chinese Academy of Medical Sciences, Beijing, 100730 China

**Keywords:** NR5A1, Mutation, Disorders of sex development, Splice, Minigene

## Abstract

**Objective:**

To identify the pathogenic mechanism of the c.244G>T mutation in *NR5A1* gene found in a Chinese patient with 46, XY disorders of sex development (DSD). Subjects and methods: Genomic DNA was extracted from a Chinese 46, XY DSD patient. Targeted next-generation and Sanger sequencing were performed to investigate and validate the gene mutation causing 46, XY DSD, respectively. In silico tools were used to predict the pathogenicity of the variant. Dual luciferase reporter gene assay and minigene splicing reporter assay were used to identify the pathogenicity of the variant.

**Results:**

A novel heterozygous variant, c.244G>T (p.Ala82Ser), in *NR5A1* gene was detected in the 46, XY DSD patient. Four of five silico tools predicting pathogenicity of missense variants indicated that the variant was pathogenic. However, in vitro functional study showed that p.Ala82Ser did not affect the transcriptional activity of NR5A1. In silico tools predicting the potential splicing loci revealed that c.244G>T led to aberrant splicing of *NR5A1* RNA. Minigene splicing reporter assay confirmed that c.244G>T resulted in the deletion of exon2 or deletion of 19 nucleotides in 3′ end of exon2.

**Conclusions:**

Mutation of c.244G>T in *NR5A1* results in 46, XY DSD by inducing abnormal splicing of *NR5A1* RNA instead of amino acid substitution of NR5A1.

## Introduction

46, XY disorders of sex development (DSD) is characterized by ambiguous external male or female genitalia with a 46, XY karyotype [1]. Depending on the etiology of the disease, 46, XY DSD is divided into disorders of gonadal development, disorders of androgen synthesis or action, and other defects, including severe hypospadias and cloacal exstrophy [2]. Chromosomal aberrations and gene mutations have been identified as the underlying causes of the disease. Currently, more than 60 genes have been linked to 46, XY DSD [3]. Specifically, mutations in *NR5A1* are detected in about 10%-20% of 46, XY DSD patients.

Nuclear receptor subfamily 5 group A member 1 (NR5A1), also known as steroidogenic factor 1 (SF1), is a member of nuclear receptor family. It plays a crucial role in transcriptional regulation of genes involved in steroidogenesis, gonadal development, and reproduction [4]. *NR5A1* gene is located on chromosome 9q33.3 and contains seven exons with one noncoding exon [5]. Mutations in *NR5A1* gene commonly cause a wide range of phenotypes, including primary adrenal deficiency, disorders of sex development (DSD), male factor infertility, primary ovarian insufficiency, and spleen anomalies [6, 7]. More than 180 mutations in *NR5A1* have been reported [7], and the most common phenotype of *NR5A1* gene mutations is 46, XY DSD.

Nowadays, gene sequence analysis has widely been used to diagnose inherited diseases, leading to the identification of thousands of rare variants. Interpreting the pathogenicity of these variants has proven to be a big challenge. Common approaches tend to explicate the clinical significance of gene variants based on the substitution of amino acids. However, recent studies have shown that exonic single-nucleotide variants can affect RNA splicing [8–10]. Hence, the effects on both the amino acid substitution and RNA splicing need to be considered during the analysis of the pathogenicity of exonic single-nucleotide variants.

Here, we reported an exonic single-nucleotide variant of *NR5A1* gene that caused 46, XY DSD by inducing abnormal splicing of *NR5A1* RNA instead of amino acid substitution.

## Results

### Patient and genetic analysis

A 16-year-old girl was referred to our hospital because of clitoris hypertrophy and primary amenorrhea. The patient was born on the due date after an uneventful pregnancy. At birth, the external genitalia showed a female phenotype. At the age of 12 years, hirsutism and deepening of the voice were noted. She was admitted to the local hospital for karyotype testing, which showed a 46, XY karyotype. For further treatment, the patient was referred to our hospital. On physical examination, the patient’s breast showed Tanner stage 1, and external genitalia showed clitoris hypertrophy with a male phenotype with pubic hair of Tanner stage 6. There was neither consanguineous marriage nor similar disease in the family.

Hormone levels were as follows: luteinizing hormone, 35.64 IU/L; follicle-stimulating hormone, 93.91 IU/L; serum testosterone, 7.74 nmol/L (reference: 6.07–27.1 nmol/L); estradiol, 73.4 pmol/L (reference: 0–172.5 pmol/L); adrenocorticotrophic hormone, 33.5 pg/mL (0–46 pg/mL); and serum cortisol, 423.4 nmol/L (reference: 110.6–616.4 nmol/L). After discussing with the parents, the patient decided to continue in a female gender and underwent laparoscopic bilateral gonadectomy. No Müllerian structure was found and bilateral gonads were located in the pelvic cavity. Histology of the left and right gonad revealed dysplastic testicular tissue.

**Fig. 1:**
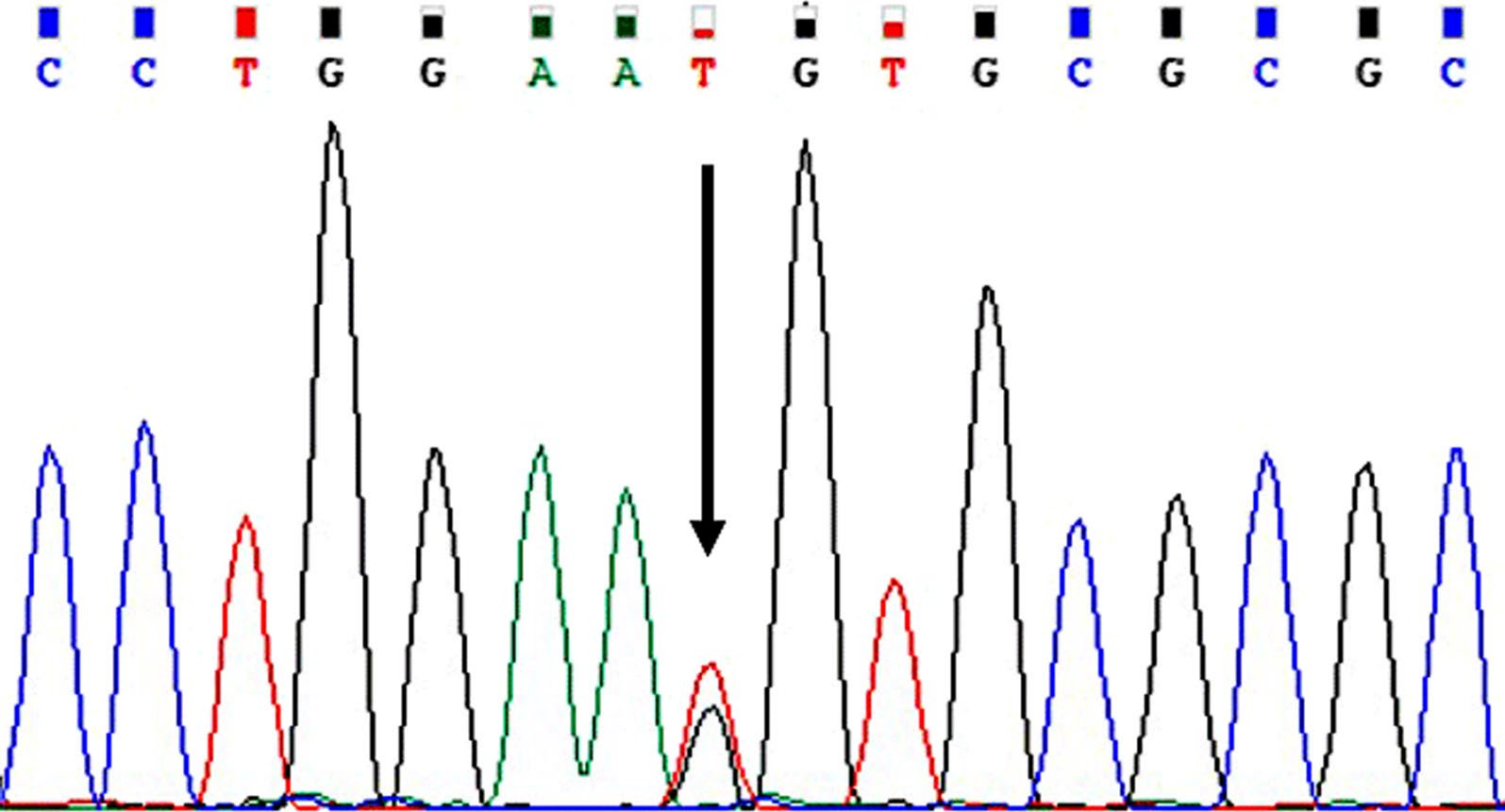
Sanger sequencing analysis of the *NR5A1* gene. The black arrow denotes the position of the mutation

Next-generation sequencing revealed a novel heterozygous *NR5A1* variant, c.244G>T, which had led to the change of the amino acid at the position 82 from Ala to Ser; Sanger sequencing confirmed the variant (Fig. 1). The variant of c.244G>T was absent in the dbSNP, ExAC, gnomAD, and Ensembl database.

### In silico analysis of *NR5A1* variants

**Fig. 2:**
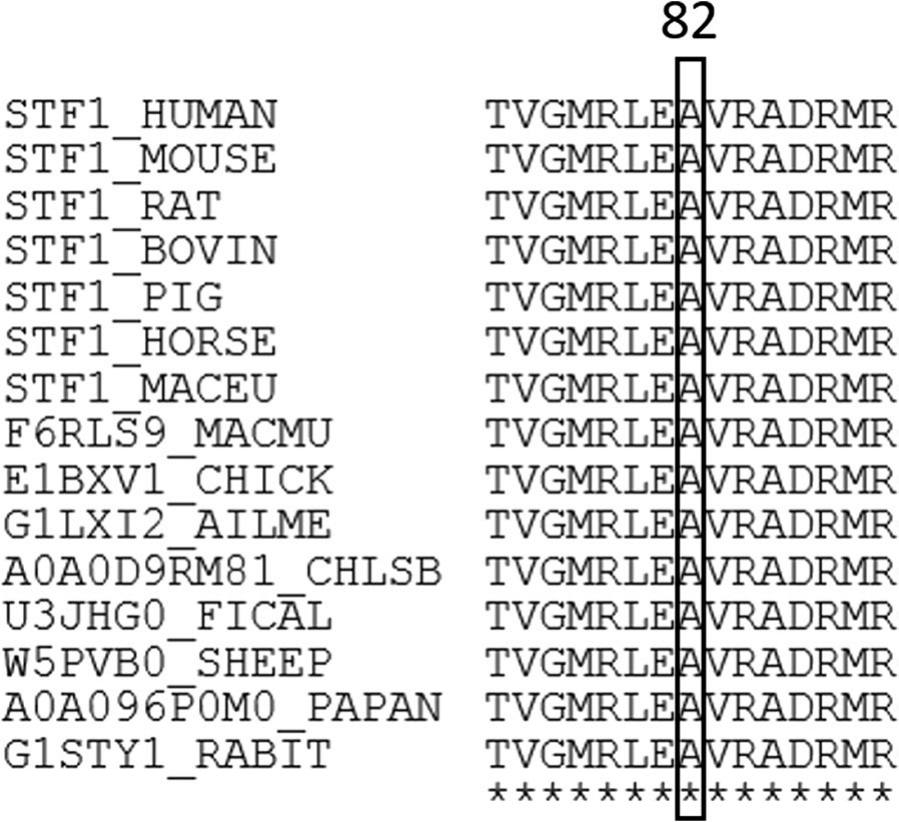
The amino acid sequence alignment of human NR5A1 with other species. The number and black box indicate the mutant residues of NR5A1

The amino acid, Ala82, is highly conserved (Fig. 2). Functional predictions of the missense mutation by Sift, SNAP, SNP&GO, Polyphen2, MutPred, and REVEL are shown in Table 1. Sift, SNAP, SNP&GO, Polyphen2, and REVEL predicted the variant as deleterious, while MutPred predicted it as a natural variant.

**Table 1:** In silico analysis of the *NR5A1* gene variants

	Software	Effect	Value		Software	Effect
Missense variant prediction	Sift SNAP SNP&GO Polyphen2 MutPred REVEL	+ + + + − +	0 30 Disease 0.998 0.485 0.787	Splicing prediction	HSF Ex skip ESE finder MaxEntScan Splice site score calculation	+ + − 9.05 → 2.86 7.6 → 4.1

In silico tools, HSF and Ex Skip, predicted that c.244G>T affected *NR5A1* RNA splicing, while ESE Finder indicted a negative result. The splicing score calculated by MaxEntScan and Splice site score calculation is shown in Table 1.

### Functional studies

#### Dual luciferase reporter gene assay

**Fig. 3:**
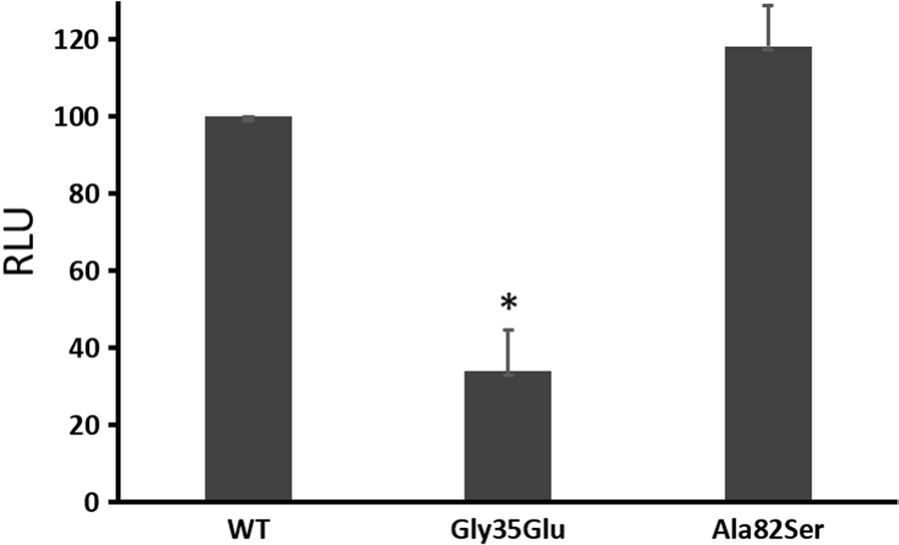
The transcriptional activity of NR5A1 mutants. The results are expressed as percent changes compared with the activity of WT-NR5A1. Error bars represent standard deviations. RLU, relative luciferase activity; WT, wild type; *P < 0.05

The transactivation activity of the c.244G>T mutants compared with that of the wild type was 118%, meaning that it was comparable to the wild type (Fig. 3), while the positive control G35E mutant showed a partial transactivation activity of 34%.

#### Minigene splicing reporter assay

**Fig. 4:**
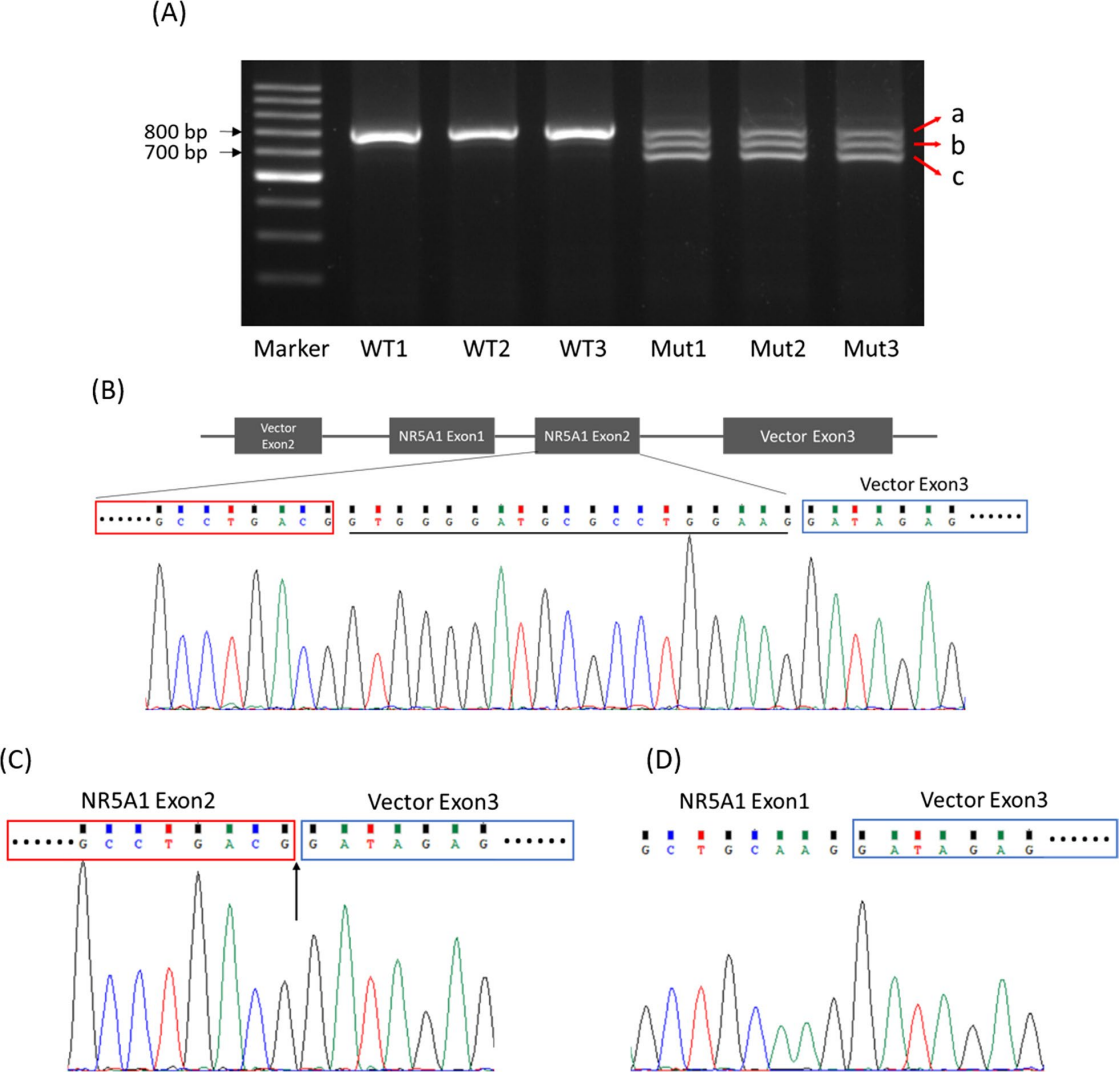
Minigene splicing reporter assay for the c.244G>T mutant. **a** RT-PCR products of minigene. 1, 2, 3 WT minigene products; 4, 5, 6 c.244G>T mutant minigene products. **b** Sanger sequencing of RT-products of WT; the line denotes the missing sequence in **c**. **c** Sanger sequencing of RT-products of the medium size RT-PCR products of c.244G>T mutant; the arrow indicates the breakpoint of the deletion sequence. **d** Sanger sequencing of RT-products of the shortest RT-PCR product of c.244G>T mutant

The RT-PCR amplification products of c.244G>T mutant in minigene showed three fragments of different lengths by electrophoresis. One was of similar length to that in the wild type, while the other two were shorter (Fig. 4a). Sanger sequencing of the longest product showed that the wild type sequence came from the normal splicing (Fig. 4b). The sequencing of the medium product showed a loss of 19 nucleotide base pairs (GTGGGGATGCGCCTGGAAG) in 3′ end of exon2 (Fig. 4c), whereas the shortest product revealed a skipping of exon2 (Fig. 4d).

## Discussion

In this study, a novel *NR5A1* variant, c.244G>T (Ala82Ser), was detected in a Chinese patient with 46, XY DSD. The Ala82 is in the A-box of NR5A1, which is essential for stabilizing DNA binding by interacting with the DNA minor groove of target genes [11]. Four of five silico tools predicted that Ala82Ser was deleterious. In contrast, in vitro functional studies showed that Ala82Ser did not affect the transcriptional activity of NR5A1 to CYP11A1. However, minigene splicing reporter assay showed that c.244G>T led to abnormal splicing.

Over the last decade, as new DNA sequencing technologies have widely been used in clinical practice, the knowledge on novel variants associated with diseases has accumulated. About 50% of these variants are missense variants [12], and clarification of the significance of rare missense variants has been difficult. Generally, bioinformatic algorithms are adopted to analyze the pathogenicity of the variants. However, previous studies have reported the false-positive and false-negative rates of the prediction tools [13–16]. Thusberg et al. [13] reported that the accuracy of Sift, SNAP, SNP&GO, Polyphen2, and MutPred was 0.65, 0.72, 0.82, 0.71, and 0.81, respectively. Zou et al. [17] reported that using Polyphen2 to predict 24 pathogenic mutations classified two of the mutations as benign. Thus, functional studies are needed to identify the pathogenicity of variants. Similarly, in our study, the prediction results of in silico tools and the results of functional studies were in disagreement. Namely, while four of five in silico tools predicted Ala82Ser as a deleterious mutation, in vitro studies showed that mutant NR5A1 (Ala82Ser) had similar transcriptional activity to CYP11A1 compared with wild-type NR5A1.

Recent studies have shown that approximately 15%-50% of exon single-nucleotide variants can affect protein function by changing RNA splicing rather than by substituting amino acid [18, 19]. Ito et al. predicted the effect of 2390 rare synonymous and missense mutations reported in the database on RNA splicing and found that 196 mutations might affect the splicing process; minigene reporter assays confirmed that 53 mutations affect splicing [20]. Thus, considering that the typical clinical manifestations of the patient and the next-generation sequencing did not reveal other suspected pathogenic mutations, we further conducted minigene splicing reporter assay. The result showed that the mutation had led to another two types of RNA splicing, resulting in the alteration of the structure of NR5A1.

The classical splice sites of human genes are located at exon–intron boundaries, including a 5′ splice donor and a 3′ splice acceptor sequence [21]. The 5′ splice donor usually contains a 9-bp sequence with a classical GT dinucleotide, while the 3′ splice acceptor contains a 23-bp sequence with an AG dinucleotide [22]. Variants occurring within the 9- or 23-bp of the splice sites have been known to interrupt normal splicing process and lead to exon skip [23]. In this study, c.244G>T was found in the 5′ splice donor sequence of the exon2 in *NR5A1*, and the minigene splicing reporter assay confirmed that c.244G>T resulted in the deletion of exon2 or deletion of 19 nucleotides in 3′ end of exon2 by disrupting the splice donor, thereby altering the structure of NR5A1.

In conclusion, mutation of c.244G>T in *NR5A1* results in 46, XY DSD by causing abnormal splicing of *NR5A1* RNA rather than substitution of amino acid.

## Patients and methods

### Patients

Patients and methods have been described in detail previously [24]. Briefly, 60 patients with 46, XY DSD were admitted to the Endocrinology Department of Peking Union Medical College Hospital, Beijing between January 2010 and October 2015. The clinical data and laboratory test results were collected in detail.

### Mutational analysis

Targeted next-generation sequencing and Sanger sequencing were used to identify and validate the gene mutation, respectively. The gene panel (NimblegenSeqCap EZ system, Roche, Basel, Switzerland) was designed to capture all exons and 50-bp flanking intron sequences of 83 DSD-related genes [24]. A variant was recognized as an underlying disease-causing variant when it was not found in dbSNP, ExAC, GnomAD, and Ensemble database and in 500 Chinese controls, or alternatively, when the allele frequency was found to be less than 0.001 in the database. The amino acid sequence alignment of human NR5A1 with that of other 14 species was performed using UniProt.

### Dual-Luciferase reporter gene assay

Mutant *NR5A1* eukaryotic expression vectors containing the c.244G>T variant and p.G35E (positive control) were generated by site-directed mutagenesis (Transgene, China). Luciferase reporter vectors comprising NR5A1-responsive promoters of human *CYP11A1* in pGL3-basic (Promega, USA) were constructed to study the effect of gene variant on the transcriptional activity of NR5A1.

Transient gene expression assays for the assessment of NR5A1 function were performed in 96-well plates with the use of human embryonic kidney (HEK) 293 T cells and a Dual-Luciferase reporter assay system (Promega, USA). Wild-type or mutant *NR5A1* expression vectors (2 ng/well) were co-transfected with reporters containing NR5A1-responsive promoters (CYP11A1) (100 ng/well). Luciferase assays were performed with the use of a luminometer reader (Berthold Centro XS3 LB 960, Germany). All data were standardized for Renilla activity, and the results are shown as mean ± standard error of mean of three independent experiments, each performed at least in triplicate.

### In silico analysis

To predict the pathogenicity of the variant, five in silico tools were used, including Sift (http://sift.jcvi.org/), SNAP (http://rostlab.org/services/snap), SNP&GO (http://snps-and-go.biocomp.unibo.it/snps-and-go), Polyphen2 (http://genetics.bwh.harvard.edu/ pph2), MutPred (http://mutpred.mutdb.org), and REVEL (https://sites.google.com/site/revelgenomics/about).

Predictions of the presence of exonic splice enhancer or silencer sequences were made using Human Splicing Finder (HSF) (http://www.umd.be/HSF3/index.html) and ESE Finder (http://krainer01.cshl.edu/cgi-bin/tools/ESE3/esefinder.cgi?process = home). Ex SKIP (http://ex-skip.img.cas.cz/) was used to determine whether the variant leads to skipping of the exon. Splice scores for the natural and cryptic donor and acceptor splice sites were determined using the MaxEntScan (http://genes.mit.edu/burgelab/maxent/Xmaxentscan_scoreseq.html) and Splice Site Score Calculation (http://rulai.cshl.edu/new_alt_exon_db2/HTML/score.html).

### Minigene splicing reporter assay

Exon1 with 125 flanking 5′ UTR bases, intron1, and exon2 with 120 flanking nucleotides of the *NR5A1* gene were amplified from the genomic DNA of the patient and a healthy control (total product size: 577 bp) by polymerase chain reaction (PCR), and then the PCR products were inserted between the BamHI and MluI restriction sites of the minigene vector to construct mutant and wild-type pCAS2 minigene vector [9], respectively.

Transfection of pCAS2 minigene constructs into the human embryonic kidney (HEK) 293 T cells was performed in 12-well plates. Forty-eight hours after the transfection, the cells were lysed and total RNA was extracted using the Total RNA Kit (OMEGA, USA). Complementary DNA (cDNA) was synthesized from 1 μg of total RNA using the PrimeScript™ RT reagent kit (Takara, Japan). PCR amplification was performed by 2 × Hieff™ PCR Master Mix (Yeasen) using the pCAS2-RT-F and pCAS2-RT-R primers. Reverse-transcription (RT) PCR products of different lengths were separated by agarose gel electrophoresis (2%), purified, and subjected to Sanger sequencing.

The sequencing results were compared with the *NR5A1* DNA sequence (NG_008176.1) and cDNA sequence (NM_004959.5) to determine whether the c.244G>T affected the splicing of *NR5A1* RNA.

## Data Availability

Not applicable.
